# Malaria and curable sexually transmitted infections in pregnant women: A two-years observational study in rural Burkina Faso

**DOI:** 10.1371/journal.pone.0242368

**Published:** 2020-11-16

**Authors:** Serge Henri Zango, Moussa Lingani, Innocent Valea, Ouindpanga Sékou Samadoulougou, Biebo Bihoun, Toussaint Rouamba, Karim Derra, Eli Rouamba, Phillipe Donnen, Michele Dramaix, Halidou Tinto, Annie Robert

**Affiliations:** 1 Pôle d’Epidémiologie et biostatistique, Institut de Recherche Expérimentale et Clinique (IREC), Université catholique de Louvain (UCLouvain), Brussels, Belgique; 2 Institut de Recherche en Sciences de la Santé, Direction Régionale du Centre Ouest (IRSS/DRCO), Bobo-Dioulasso, Burkina Faso; 3 Centre MURAZ, Institut National de Santé Publique (INSP), Bobo-Dioulasso, Burkina Faso; 4 École de santé publique, Université Libre de Bruxelles, Bruxelles, Belgique; Instituto Rene Rachou, BRAZIL

## Abstract

**Background:**

Malaria and curable sexually transmitted infections (STI) are the most common curable infections known to have a severe impact on pregnancy outcomes in sub-Saharan Africa. This study aims to assess the marginal and joint prevalence of symptomatic cases of malaria and STI in pregnant women living in rural settings of Burkina Faso and their associated factors, after more than a decade of the introduction of intermittent preventive treatment (IPT-SP).

**Methods:**

We carried out an observational study in two health districts in rural Burkina, namely Nanoro and Yako. Routine data were collected during antenatal and delivery visits for all women who delivered in the year 2016 and 2017. Logistic regression models were used to assess factors associated with infections.

**Results:**

We collected data from 31639 pregnant women attending health facilities. Malaria, curable STI and their coinfections were diagnosed in 7747 (24.5%; 95%CI: 24.0–25.0%), 1269 (4.0%; 95%CI: 3.8–4.2%) and 388 (1.2%; 95%CI: 1.1–1.4%) women, respectively. In multivariate logistic regression, malaria occurrence was significantly higher in pregnant women < 20 years (Adjusted OR = 2.36; 95% CI: 2.07–2.69) than in women ≥30 years. The prevalence of curable STI was also significantly higher in students (Adjusted OR = 1.93; 95% CI: 1.26–2.95) and compensated workers (Adjusted OR = 1.52; 95% CI: 1.01–2.17) than in uncompensated workers. Women who received no IPT-SP had higher prevalence of malaria (Adjusted OR = 3.33; 95%CI: 3.00–3.70), curable STI (Adjusted OR = 1.96 95%CI: 1.60–2.39) and coinfections (Adjusted OR = 2.11; 95% CI: 1.50–2.95) compared to women who received SP.

**Conclusion:**

Malaria and curable STI remain highly prevalent in rural settings of Burkina Faso, with young pregnant women and women who received no IPT-SP being the most affected. Prevention must be reinforced to improve maternal and infant health.

## Introduction

Malaria and curable sexually transmitted infections (STI), namely syphilis, gonorrhea, trichomoniasis, and chlamydia are the most common curable infections responsible for poor outcomes of pregnancy in sub-Saharan Africa [1,2]. These adverse outcomes include low birth weight, prematurity, stillbirth, and miscarriages [3–7]. In Burkina Faso, as in most of sub-Saharan countries, two main interventions are implemented to prevent malaria in pregnant women: long-lasting insecticide-treated nets to prevent the biting of the *Anopheles* mosquitoes, and intermittent preventive treatment with sulfadoxine-pyrimethamine (IPT-SP) according to recommendations from the World Health Organisation (WHO) [8,9]. Also, effective management of malaria cases detected during antenatal visits with a rapid diagnostic test (RDT) or blood smear microscopy and effective treatment with the artemisinin-based combination could contribute to reducing the burden of malaria [10]. Nevertheless, many cases of malaria are not managed due to the low performance of different malaria diagnostic methods. Indeed, authors report that the *Plasmodium* lactate dehydrogenase-based RDT (widely used in the routine) and microscopy have a low sensitivity: 54.8% (48–62%) and 54.2% (47–61%) respectively, but a good specificity: 97.9% (95–99%) and 98.3% (95–100%) respectively, when compared to polymerase chain reaction as reference test [11]. Case management of STI in pregnant women is the main strategy to reduce their impact on birth outcomes in Burkina Faso. However, among various curable STIs, only syphilis is systematically screened in pregnant women using a rapid diagnostic test. The other STIs are clinically diagnosed and treated during the antenatal visit [12]. The use of syndromic management as a diagnostic method for curable STIs in the routine care remains widely known as an insufficient diagnostic method with a low sensitivity (30–80%) and specificity (40–80%) for gonorrhea and chlamydia and trichomoniasis among pregnant women [13–15]. So, the majority of curable STIs are not clinically diagnosed.

Information on cases are routinely collected in the health system information. In 2006, when the IPT-SP strategy was adopted, some authors reported malaria incidence rate at 39.2 per 1,000 pregnant women-months in the rural health facilities of Boucle du Mouhoun region in Burkina Faso [16]. In 2014, just before the revision of the strategy from two doses to at least three doses of IPT-SP during pregnancy, the prevalence of malaria was 27% in pregnant women in Nanoro, Burkina Faso [9]. Since 2003 with a reported seroprevalence of syphilis of 1.7% [17], only the prevalence of *Trichomonas vaginalis* (13%) in teenage pregnant women was published among curable STI in pregnant women population in rural setting of Burkina Faso [18]. We herein report the results of an observational study carried out in the hand-recorded registries of two health districts in rural Burkina Faso, namely Nanoro and Yako. We aimed to provide estimates of the health facility-based prevalence of symptomatic malaria and curable STI detected by symptoms in pregnant women and associated maternal characteristics. Malaria and curable STI co-infections were also explored in these rural settings.

## Materials and methods

### Study area

**Fig 1:**
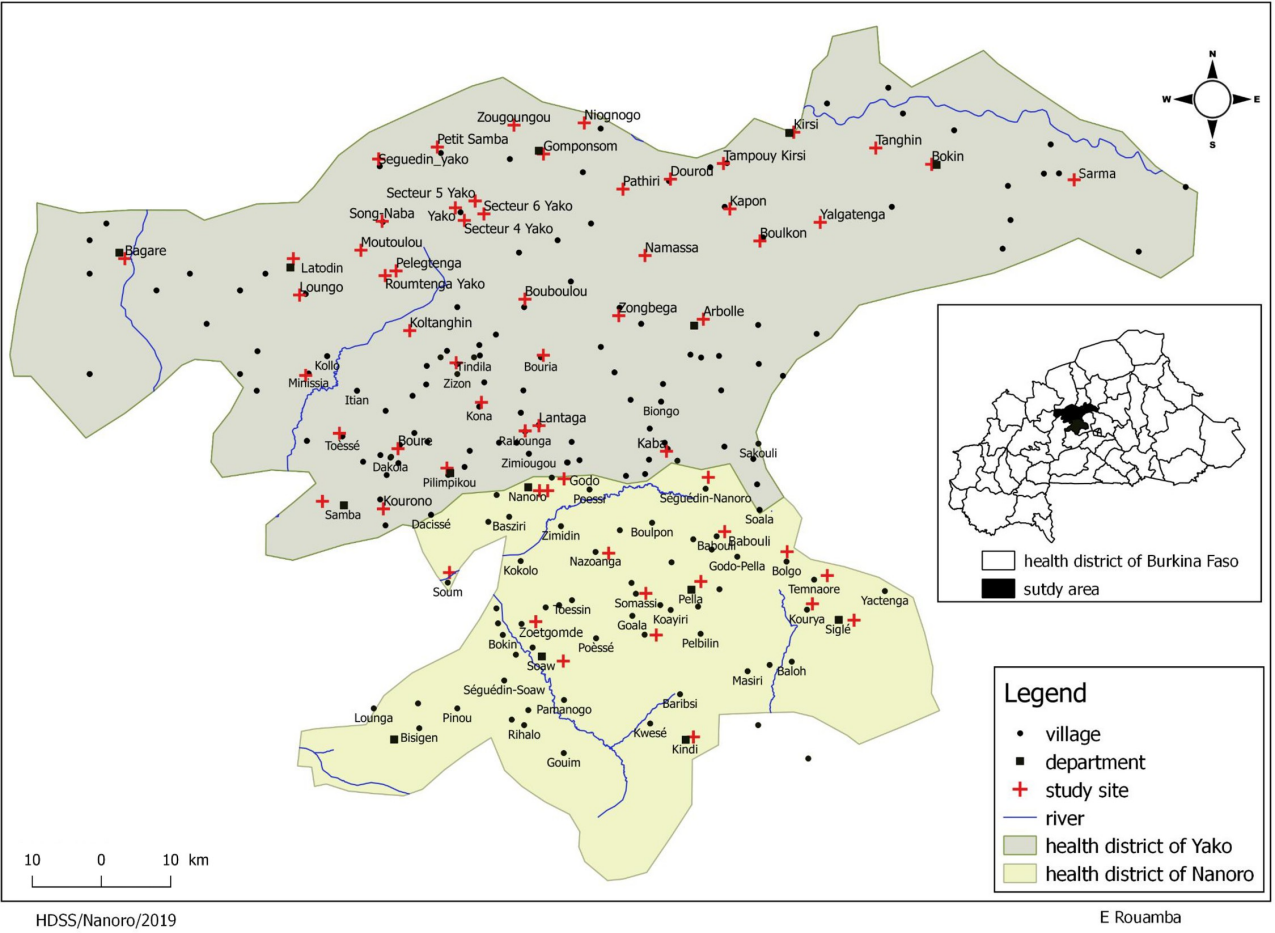
Map showing Yako and Nanoro Health Districts area. Health facilities were localized using Global positioning (GPS). Shapefile downloaded from https://www.diva-gis.org/ Created by Eli Rouamba, 2019.

The study was carried out in both Nanoro health district (NHD) and Yako health district (YHD) which are neighboring health districts, with NHD in the south of YHD (Fig 1). In 2016, both districts had 77 health facilities covering a total population of 580.279 inhabitants, 166.683 in NHD, and 413.596 in YHD. The number of expected pregnancies based on national annual statistics was 8957 and 22032 in NHD and YHD, respectively. So, the total expected number of pregnancies was 30989 [19]. In this area, malaria transmission is highly seasonal and overlaps with the rainy season that lasts from July to December [20].

### Study design and data collection

We conducted a retrospective study in 61 out of 77 health facilities belonging to NHD and YHD. Sixteen health facilities were excluded because they were too far or because of a lack of hand-recorded registries. In the routine health system, data of pregnant women must be collected in hand-recorded registries by the nurses and midwives during the antenatal care visits and at delivery visits.

### Study population

We considered data from all women who delivered between January 2016 and December 2017 in this study. The data of ANC visits performed in 2015 for women who delivered at the beginning of 2016 were also considered. The data were captured retrospectively during the last trimester of 2018 in electronic pads using the REDCap application.

### Definition of terms

In rural health settings, the diagnosis of malaria and that of STI are performed according to the national guidelines. Based on clinical signs or history of fever, all suspected cases of malaria in pregnant women are investigated by using malaria RDT or a microscopy examination. The confirmed cases of uncomplicated malaria are treated with artemisinin-based combination (artemether-lumefantrine or artesunate-amodiaquine), or quinine. Also, all malaria cases diagnosed in surveys in the area are transferred to health facilities for the treatment. A systematic screening of syphilis is performed for all pregnant women at their first ANC visit. The diagnostic of other curable STI is carried out using clinical syndrome-based algorithms as recommended by the WHO for the constrained resource setting [21]. The marginal prevalence of malaria or curable STI is the proportion of women infected by each type of infection among women attending the health facilities. The joint prevalence is the proportion of the co-infections of both malaria and curable STI. We defined co-infection as the occurrence of both malaria and curable STI in a pregnant woman during the same pregnancy. Student means that the pregnant woman was continuing secondary or higher education. Women with compensated work means that the pregnant women had an outdoor remunerative activity (civil servant, market vendor, hairdresser). Women with uncompensated work are usually farmers who produce for family consumption. Primigravidae means that the women were in their first pregnancy, secundigravidae for those that were in their second pregnancy, and multigravidae were used for women in their third pregnancy or more. The history of miscarriage was defined as a fetal death before 28.0 weeks of gestation that occurred in a prior pregnancy. The history of stillbirth was defined as delivery of a new-born with no signs of life between 28.0 and 37.0 weeks of gestation in a prior pregnancy. Female genital mutilation (FGM) was defined as partial or total removal of the external female genitalia.

### Statistical analysis

All analyses were performed with Stata^®^ version 15.1. The statistical significance level was set to 0.05. We included all women having at least one visit in the period to compute prevalence. Cochran-Armitage test was used to assess the trends in the prevalence of malaria, curable STI, and co-infection by year. Complete cases mean all cases not having missing data for the covariates used in the models. Univariate and multivariate logistic regressions were performed to identify factors associated with malaria and STIs. Before performing multivariate regression analysis, correlation coefficients were checked for multicollinearity, and variance inflation factors were used for eliminating a covariate if greater than 10. Only factors associated (with a p-value < 0.05) with at least one of them in the univariate logistic regression analysis were considered in multivariate regression. Odds ratio and adjusted odds ratio are reported with their 95% confidence intervals.

### Ethical considerations

Ethical approval for this study was obtained from both the « Comité d’Ethique Hospitalo-Facultaire Saint-Luc UCLouvain » in Belgium and the National Ethics Committee (Comité d’Ethique pour la Recherche en Santé, CERS) in Burkina Faso. The identities of all patients were anonymized during the data capture.

## Results

**Fig 2:**
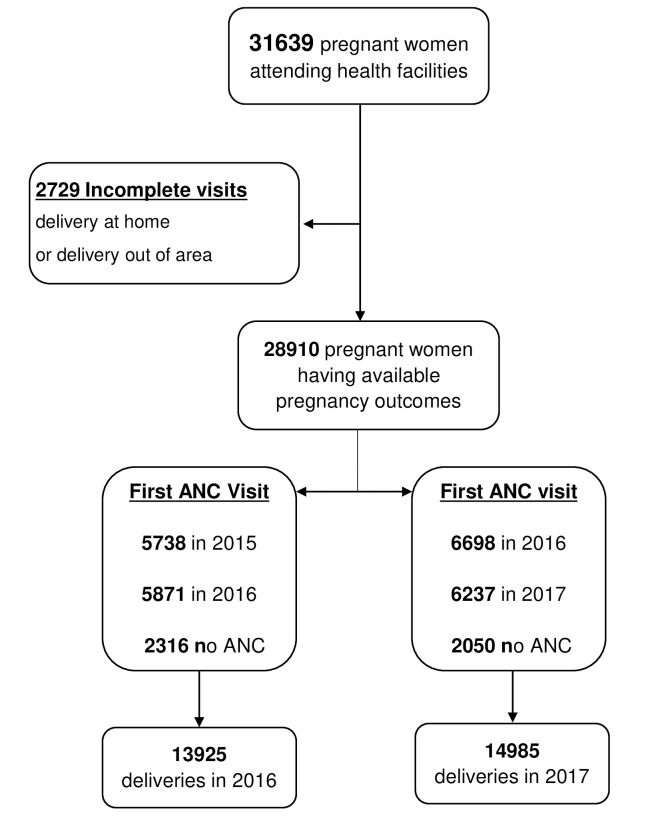
Flow chart of patients showing visit performed and selection flow by year.

Overall, data from 31639 pregnant women were collected in 61 health facilities (Fig 1). The number of women with missing delivery visit was 2729 (8.6%) (Fig 2). The mean age of women was 25.6 years, including 19.8% of teenagers, 94.6% of them were women with uncompensated work and 2.2% were students. Multigravidae were more represented (44.9%) (Table 1). Sixty-two pregnant women (0.2%) were HIV seropositive.

**Table 1:** General characteristics of the study population.

	Available data			Complete cases (n = 17826)	
	n	Mean ± SD	%	Mean ± SD	%
Age at entry (years)	26672	25.4 ± 6.3		25.6 ± 6.3	
< 20			20.9		19.8
20 to 29			51.9		52.0
≥ 30			27.2		28.2
Occupation	28539				
Uncompensated workers			94.2		94.6
Student			2.2		2.0
Compensated workers			3.6		3.4
Married	23605				
Yes			98.2		98.5
No			1.8		1.5
Gravidity	27678				
Primigravidae			22.7		21.8
Secundigravidae			33.6		33.3
Multigravidae			43.7		44.9
History of miscarriage	29801				
Yes			2.0		2.2
No			98.0		97.8
History of stillbirth	29801				
Yes			0.5		0.6
No			99.5		99.4
Female genital mutilation	29801				
Yes			42.1		46.6
No			57.9		53.4
IPT-SP^a^	20861				
Yes			14.2		11.8
No			86.8		88.0

^a^IPT-SP: Intermittent preventive treatment with sulfadoxine-pyrimethamine in pregnant women.

Between August 2015 to December 2017, symptomatic malaria, curable STI detected by symptoms and co-infections were diagnosed in 7747 (24.5%; 95%CI: 24.0–25.0%), 1269 (4.0%; 95%CI: 3.8–4.2%) and 388 (1.2%; 95%CI: 1.1–1.4%) pregnant women, respectively. Among women having experienced malaria, 960 (3.0%; 95%CI: 2.9–3.2%) and 197 (0.6%; 95%CI: 0.5–0.7%) had two and three episodes, respectively. Two episodes of curable STI were reported in 154 (0.5%; 95%CI: 0.4–0.6%) pregnant women. Table 2 describes prevalence of these infections and shows an increase from 2015 to 2017 in prevalence of symptomatic malaria, curable STI detected by symptoms, and of co-infections.

**Table 2:** Health facility-based prevalence of symptomatic malaria and curable STI.

	Prevalences (%)				
	Overall	2015	2016	2017	Cochran X^2^ P-value
	N = 31639	N = 5738	N = 15852	N = 10049	
Malaria	24.5	21.4	21.9	30.4	<0.001
STI^a^	4.0	3.3	3.5	5.2	<0.001
Co-infections^b^	1.2	0.9	1.1	1.6	<0.001

^a^STI: Sexually transmitted infections.

^b^Cases included in both previous lines.

Table 3 reports the univariate and multivariate associations of women characteristics with malaria. The prevalence of symptomatic malaria significantly decreased with the age of the pregnant women. It was also significantly higher in the students (Adjusted OR = 1.38; 95% CI: 1.06–1.79) than in women with uncompensated work, after adjusting to other covariates.

**Table 3:** Factors associated with malarial infection in univariate and multivariate logistic regression analyses in pregnant women.

	n	Malaria %	OR (95%CI)	Adj OR (95%CI)
**Age (years)**				
< 20	3524	20.2	2.62 (2.31–2.98)**	2.36 (2.07–2.69)**
20 to 29	9279	13.2	1.58 (1.41–1.77)**	1.53 (1.36–1.71)**
≥30	5023	8.8	1.00	1.00
**Occupation**				
Students	362	22.9	1.97 (1.54–2.53)**	1.38 (1.06–1.79)*
Compensated workers	599	15.5	1.22 (0.97–1.53)	1.07 (0.86–1.34)
Uncompensated workers	16865	13.1	1.00	1.00
**Married**				
Yes	17552	13.3	0.76 (0.55–1.05)	NI
No	274	16.8	1.00	
**Gravidity**				
Primigravidae	3885	16.5	1.50 (1.34–1.67)**	1.12 (0.99–1.26)
Secundigravidae	5938	13.7	1.20 (1.09–1.33)**	1.07 (0.97–1.19)
Multigravidae	8003	11.7	1.0	1.00
**History of miscarriage**				
Yes	394	12.2	0.90 (0.66–1.22)	NI
No	17432	13.4	1.00	
**History of stillbirth**				
Yes	100	15.0	1.14 (0.66–1.98)	NI
No	17726	13.4	1.00	
**Female genital mutilation**				
Yes	8313	12.8	0.91 (0.83–0.99)*	0.92 (0.84–1.01)
No	9513	13.9	1.00	1.0
**IPT-SP**				
Yes	15716	11.2	1.00	1.00
No	2110	29.8	3.38 (3.04–3.76)**	3.33 (3.00–3.70)**

IPT-SP: Intermittent preventive treatment with sulfadoxine-pyrimethamine in pregnant women; OR: Odds ratio; Adj OR: Adjusted odds ratio

*P-value < 0.05

**p-value < 0.001; NI: Not included.

The prevalence of symptomatic malaria was significantly higher in primigravidae and secundigravidae than in multigravidae in univariate analysis, but these associations did not remain in multivariate analysis. Women who received no IPT-SP experienced significantly more malaria compared to women who received IPT-SP (Adjusted OR = 3.32; 95%CI: 2.96–3.74).

All variance inflation factors were lower than 10, so no multicollinearity was observed; the greater correlation was +0.27 (variance inflation factors = 1.1), corresponding to a positive association between age and gravidity, a mark of reliability.

History of miscarriage, stillbirth, or FGM was not associated with malaria infections in univariate regression.

Table 4 reports univariate and multivariate associations of women characteristics with curable STI detected by symptoms. Curable STI prevalence was higher in pregnant women aged <20 years than in women aged over 30 years in univariate analysis but this association did not remain in multivariate. Both women with compensated work and students experienced more curable STI than women with uncompensated work in univariate and multivariate analysis. Women having an FGM significantly experienced less curable STI and the reduced prevalence remained after adjustment (Adjusted OR = 0.76; 95%CI: 0.64–0.90). Women who received no IPT-SP experienced more STI than women who received IPT-SP (Adjusted OR = 1.96; 95%CI: 1.60–2.39).

**Table 4:** Factors associated with curable STI detected by symptoms in univariate and multivariate logistic regression analyses in pregnant women.

	n	STI %	OR (95%CI)	Adj OR (95%CI)
**Age in years**				
< 20	3524	3.8	1.27 (1.00–1.62)*	1.14 (0.90–1.46)
20 to 29	9279	3.3	1.12 (0.92–1.36)	1.11 (0.91–1.35)
≥30	5023	3.0	1.00	1.00
**Occupation**				
Students	362	7.2	2.36 (1.58–3.57)**	1.93 (1.26–2.95)*
Compensated workers	599	5.7	1.85 (1.29–2.64)**	1.52 (1.07–2.17)*
Uncompensated workers	16865	3.2	1.00	1.00
**Married**				
Yes	17552	3.3	0.91 (0.48–1.71)	NI
No	274	3.7	1.00	
**Gravidity**				
Primigravidae	3885	4.0	1.35 (1.10–1.66)*	1.19 (0.96–1.49)
Secundigravidae	5938	3.4	1.15 (0.95–1.39)	1.12 (0.92–1.35)
Multigravidae	8003	3.0	1.00	1.00
**History of miscarriage**				
Yes	394	5.3	1.66 (1.06–2.60)*	1.56 (0.98–2.46)
No	17432	3.3	1.00	1.00
**History of stillbirth**				
Yes	100	4.0	1.21 (0.45–3.31)	NI
No	17726	3.3	1.00	
**Female genital mutilation**				
Yes	8313	2.8	0.72 (0.61–0.86)**	0.76 (0.64–0.90)*
No	9513	3.8	1.00	1.00
**IPT-SP**				
Yes	15716	3.0	1.00	1.00
No	2110	6.0	2.08 (1.70–2.54)**	1.96 (1.60–2.39)**

STI: Sexually transmitted infections; NI: Not included; Adj OR: Adjusted odds ratio; IPT-SP: Intermittent preventive treatment with sulfadoxine-pyrimethamine in pregnant women

*p-value < 0.05

**p-value < 0.001.

**Fig 3:**
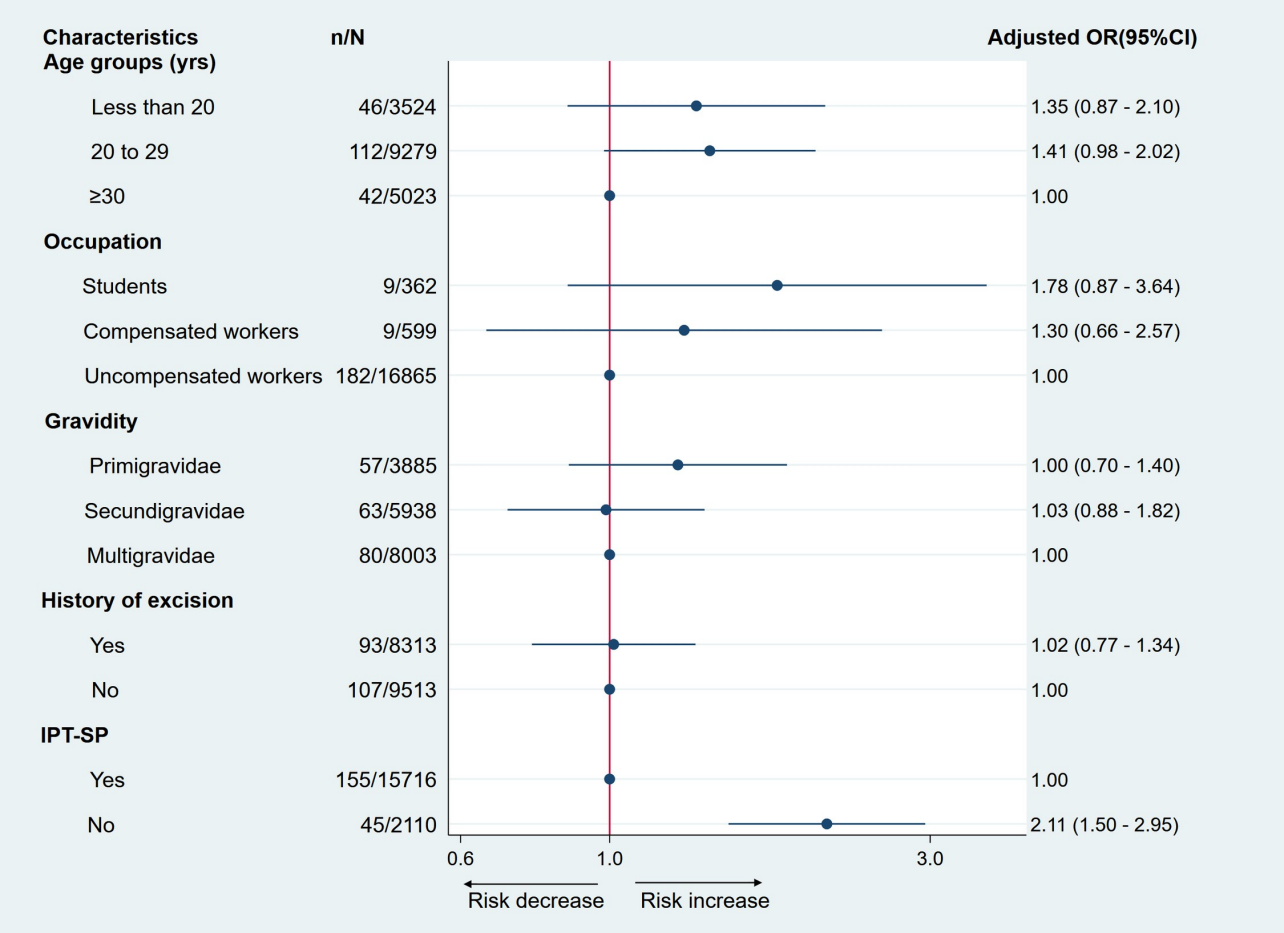
Multivariate odds ratio of coinfection malaria and curable sexually transmitted infections in pregnant women. The red solid vertical line is the reference line. Black dots are the estimated odds ratio. Horizontal black solid lines are 95% confidence intervals. On the right side of the red line, the risk of coinfection increases while it decreases on the left side. An estimate is not significant when its confidence interval crosses the red line. IPT-SP: intermittent preventive treatment with sulfadoxine-pyrimethamine.

A similar association was found in women with coinfection malaria and STI (Adjusted OR = 2.11; 95%CI: 1.50–2.95) (Fig 3).

## Discussion

The current study aimed to estimate the prevalence of symptomatic malaria and curable STI detected by symptoms in pregnant women attending rural health facilities. Information were captured retrospectively from routine data collected in a rural setting. Comparison between distributions in both available data and complete case data shows that there is no bias to use complete case data for analysis. Health facilities included in our study cover the entire study area, and a half (51%) of the number of pregnancies expected for the two-years period of deliveries. Also, the high proportion of multigravidae and women with compensated work are in concordance with rural women characteristics in Burkina Faso, reinforcing the representativity of the sample [19].

The results showed that the prevalence of symptomatic malaria between 2015 and 2017 in pregnant women was lower than reported in 2014 in Nanoro (27%) [9], or 2006 [16]. Our study used mainly symptomatic cases of malaria diagnosed during antenatal visits and some malaria cases (asymptomatic or symptomatic) screened in clinical studies or surveys in the area and transferred to the health facilities. In addition, this lower prevalence could also be explained by the reinforcement in malaria prevention in pregnant women by the introduction of the use of two doses of sulfadoxine-pyrimethamine in 2006, and the increase to at least three doses in 2014 in Burkina Faso [9]. However, malaria prevalence increased significantly from 2015 to 2017. This increase in the health facility-based prevalence of malaria in these last years has also been reported by the WHO African region [22]. In Burkina Faso, a full free maternal healthcare was implemented in 2016 in all public healthcare facilities, and the numbers of health facilities and health providers were increased [19]. This context improved the access to healthcare and increased health facilities attending that could explain the high report of diseases in health facilities. In our study, the prevalence of curable STI in pregnancy detected by symptoms was lower than the one reported for globally sub-Saharan Africa that was estimated at least 15% [3]. Many reasons could explain this low prevalence in pregnant women population in our country. Beyond the medium sensitivity and specificity of syndromic-based to diagnose gonococcal and chlamydia infections[23–25], the use of azithromycin in mass drug administration in the general population through implementation of trachoma control program [26], has certainly reduced the prevalence of curable bacterial STI such as Chlamydia, as reported for other countries [27]. The results showed a relatively low prevalence of malaria- and curable STI co-infections (1.2%), and so a high cumulated prevalence (27.3%) of both curable infections in pregnant women. These findings from routine data analysis showed that more effort must be done to reduce the prevalence of these curable infections.

For both malaria and STI, younger women (< 20 years) and the students had the highest prevalence of infection in univariate analysis, but the association did not remain after adjustment with covariates for STI. Several authors have reported a young age as a risk factor of malaria [28–33], and STI [34–36] occurrence. However, some others have not found a statistically significant relationship [37,38]. Young age is known to be a factor of the immaturity of acquired immunity against infections. In addition, the students have a higher level of exposure to mosquito bites due to lessons learning, before going to bed under the mosquito net. It is the same for young women in general, who are more exposed to mosquito bites in the evening because they go to bed later than the older ones. These habits could also explain the higher proportion of infections in these groups even after adjusting with IPT-SP and other covariates. For STI, young age is also known to be a risk factor due to high sexual behaviour at risk in this age group [21].

Gravidity did not remain statistically associated with malaria or STI after adjusting with other covariates, but a gradient was observed. The women who experienced FGM had fewer STI than those not having. Authors reported psychological psychosexual disorders caused by FGM that could lead to decreased sexual desire [39–41]. In consequence, the sexual experience decrease, and probably the number of partners, so STI risk factors [42]. In addition, other authors reported that the women who have experienced FGM are generally from a conservative family environment that could contribute to reducing these risk factors of STI [43]. Our study found a high proportion of women with FGM (46.6%), which is a notable factor in the low prevalence of STI in a rural setting.

Women who received IPT-SP were more protected against malaria, curable STI, and their coinfections. These findings showed that despite the growing sulfadoxine-pyrimethamine resistance reported by many authors in the last decade [44–47], IPT-SP remained an important strategy for malaria control in Burkina Faso. The proportion of women who received no IPT-SP was still high (11.8%). More effort could be done to get better coverage of pregnant women. The low prevalence of curable STI in women having received SP in prevention is due to the antibacterial property of SP [48,49]. Some authors investigated different preventive ways in adjoining azithromycin to the IPT-SP scheme to reduce these infections [50–52]. Azithromycin could reinforce the action of sulfadoxine-pyrimethamine in malaria prevention [53,54] and could clear asymptomatic STIs that are not routinely diagnosed in health facilities of countries with constrained resource settings [55]. In considering the difficulties to intervene at a young age that was the strongest but non-modifiable factor associated with a high prevalence, this strategy adjoining azithromycin to IPT-SP could be more adapted in the setting of constrained resource to control these infections. Nevertheless, more investigation on this strategy could be done in trials with robust design in several areas with different epidemiological profiles of malaria and STI, to bring more information about their efficacy in the prevention of infections and safety on pregnancy outcomes.

Missing data were important in this study, but there was no evidence against a missing at random hypothesis with a low impact on findings. In addition, the large size of our dataset improved the precision of estimations. The method of diagnosis using syndrome-based algorithms in the routine system can induce a misrepresentation of prevalence of symptomatic STIs and did not allow us to know the etiology of STIs. The RDTs and the microscopy have a low sensitivity that could understate the prevalence of malaria cases. Nevertheless, our results are presenting a good state of reality in malaria and STI management in rural health facilities in Burkina Faso. The large sample in our study cannot exclude that a pregnant woman has been seen twice in ANC. The number of doses of IPT-SP was not used in our study to assess the efficacy of different doses in malaria prevention because of the low reliability of this information due to the women's references for seeking the best care in another health facility. The sexual subjects are still taboo in the study area, so we have not presented a comparison between the two districts to avoid discrimination between the communities.

## Conclusion

Symptomatic malaria and curable STI decreased in the last decade in rural Burkina Faso. These severe and curable infections, known to cause poor outcomes of pregnancy, remain high in rural settings of Burkina Faso, despite implemented control interventions. Their co-infections are also frequent. Pregnant women who received no IPT-SP were the most infected. The IPT-SP is a program implemented in Burkina Faso and that could be improved by the adjunction of azithromycin to reduce adverse pregnancy outcomes related to malaria and curable infections. Young pregnant women under 20 years and students are also more frequently infected. Prevention programs targeting these subgroups of pregnant women could be developed to improve maternal and child health in resource-limited settings.

## Supporting information

S1 Table Factors associated with malarial infection in univariate and multivariate logistic regression analyses in pregnant women using complete cases and available data. (XLSX)

S2 Table Factors associated with curable STI in univariate and multivariate logistic regression analyses in pregnant women using complete cases and available data. (XLSX)

S1 Dataset (DTA)
